# Supplementary figures and images for: Automated mapping of Portulacaria afra canopies for restoration monitoring with convolutional neural networks and heterogeneous unmanned aerial vehicle imagery
Source: PeerJ. 2022 Oct 14;10:e14219. doi: 10.7717/peerj.14219 (PMC9575683; doi:10.7717/peerj.14219)

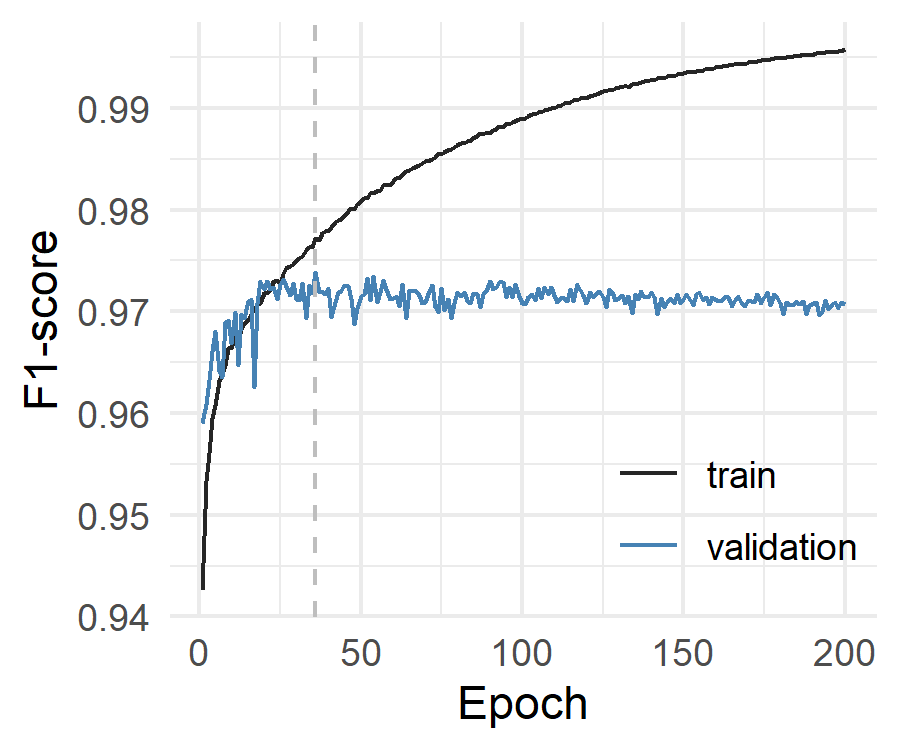

Supplement: Supplemental Information 1 — The vertical dashed line indicates the model selected for final prediction. [file peerj-10-14219-s001.png]
